# An authentic animal model of the very preterm infant on nasal continuous positive airway pressure

**DOI:** 10.1186/s40635-015-0051-4

**Published:** 2015-04-29

**Authors:** Peter A Dargaville, Anna Lavizzari, Priscila Padoin, Don Black, Elroy Zonneveld, Elizabeth Perkins, Magdy Sourial, Anushi E Rajapaksa, Peter G Davis, Stuart B Hooper, Timothy JM Moss, Graeme R Polglase, David G Tingay

**Affiliations:** Department of Paediatrics, Royal Hobart Hospital, 48 Liverpool St, Hobart, Tasmania 7000 Australia; Neonatal Research, Murdoch Childrens Research Institute, Melbourne, Victoria Australia; Menzies Institute for Medical Research, University of Tasmania, Hobart, Tasmania Australia; NICU, Fondazione IRCCS Ca’ Granda, Ospedale Maggiore Policlinico-Università degli Studi di Milano, Milan, Italy; Pontifícia Universidade Católica do Rio Grande do Sul, Porto Alegre, Brazil; Newborn Research, The Royal Women’s Hospital, Melbourne, Victoria Australia; The Ritchie Centre, Monash Institute of Medical Research and Department of Obstetrics and Gynaecology, Monash University, Melbourne, Victoria Australia; Neonatal Unit, Royal Children’s Hospital, Melbourne, Australia

**Keywords:** Preterm lamb, Continuous positive airway pressure, Non-invasive positive pressure ventilation, Apnoea

## Abstract

**Background:**

The surge in uptake of nasal continuous positive airway pressure (CPAP) for respiratory support in preterm infants has occurred in the absence of an authentic animal model. Such a model would allow investigation of research questions of physiological and therapeutic importance. We therefore aimed to develop a preterm lamb model of the non-intubated very preterm infant on CPAP.

**Methods:**

After staged exteriorisation and instrumentation, preterm lambs were delivered from anaesthetised ewes at 131 to 133 days gestation. Via a single nasal prong (4-mm internal diameter, 6- to 7-cm depth), positive pressure was delivered from the outset, with nasal intermittent positive pressure ventilation (NIPPV) used until transition to nasal CPAP was attempted, and periodically thereafter for hypoventilation. Caffeine and doxapram were used as respiratory stimulants. Gastric distension was prevented with an oesophageal balloon. Cardiorespiratory parameters and results of arterial blood gas analyses were monitored throughout the study period, which continued for 150 min after first transition to CPAP.

**Results:**

Ten preterm lambs were studied, at gestation 132 ± 1 days (mean ± SD) and birth weight 3.6 ± 0.45 kg. After stabilisation on NIPPV, transition to nasal CPAP was first attempted at 28 ± 11 min. There was transient respiratory acidosis, with gradual resolution as spontaneous respiratory activity increased. In the final hour, 79% ± 33% of time was spent on CPAP alone, with typical respiratory rates around 60 breaths per minute. PaCO_2_ at end-experiment was 58 ± 36 mmHg.

**Conclusions:**

Non-intubated preterm lambs can be effectively transitioned to nasal CPAP soon after birth. This animal model will be valuable for further research.

## Background

Unlike many areas of intensive care practice, the surge in uptake of nasal continuous positive airway pressure (CPAP) for respiratory support from birth in very preterm infants <32 weeks gestation has occurred in the absence of an authentic, robust and accessible animal model. Such a model would allow the conduct of experimental studies that are technically challenging and/or ethically unsound in human infants, and potentially give a better understanding of such issues as the effect of alteration of CPAP pressure, the relative merits of CPAP delivery devices and the optimal approach to nasal intermittent positive pressure ventilation (NIPPV). The physiological effects of adjunctive therapies applied in infants on CPAP, including recruitment manoeuvres [1], surfactant therapy [2,3] and caffeine [4,5], could also be more fully and systematically evaluated.

In the past, experimental studies of CPAP in preterm animals have been undertaken at from birth with an endotracheal tube *in situ* [6-9] or many hours after birth at a time when some of the unique physiological features of the cardiorespiratory transition at birth have dissipated [10,11]. Rahmel and co-workers recently described the first authentic animal model of nasal CPAP applied from birth, using short binasal prongs in newborn preterm lambs at 136 days gestation [12]. Their study demonstrates that CPAP delivery via a nasal interface is possible in preterm lambs in the immediate newborn period and can be used for therapeutic trials. The model has the limitations of the need for spinal anaesthesia in the pregnant ewe, the relatively mature gestation, the use of a nasal CPAP interface that may be difficult to maintain in position and a 20% mortality risk from respiratory failure within the first 30 min [12].

We wished to develop a robust model of nasal CPAP in preterm lambs delivered at a less mature gestation (131 to 133 days) from ewes receiving general anaesthesia. We aimed to evaluate whether, with a practical and effective CPAP delivery interface and therapy to induce spontaneous breathing, nasal CPAP could be applied in the immediate newborn period and produce adequate tidal ventilation and gas exchange.

## Methods

### Experimental approach

Studies were conducted within the animal research facility at the Murdoch Childrens Research Institute. The study protocol was approved by the Murdoch Childrens Research Institute Animal Ethics Committee, and the experiments conducted according to the Australian Code for the Care and Use of Animals for Scientific Purposes 2013. Date-mated Border-Leicester/Merino ewes were treated with betamethasone (11.4 mg by intramuscular injection) 48 and 24 h prior to delivery. On the day of the experiment, ewes were anaesthetised, intubated and ventilated, and venous and arterial catheters were inserted. Anaesthesia was maintained with intravenous propofol and nitrous oxide, along with isoflurane, which was used at the lowest possible concentration, and turned off for 20 min prior to delivery. In the case of twin gestation, anaesthesia was maintained for 2 to 3 h during which each fetus was sequentially instrumented and delivered. After hysterotomy, the fetal head and neck was delivered, and arterial and venous catheters were placed in the neck vessels using a cutdown technique with local anaesthesia. The fetal head was dried, and 10% lignocaine (Xylocaine spray, AstraZeneca, North Ryde, Australia) was introduced into both nostrils and applied to the vocal cords under direct laryngoscopic vision.

**Figure 1:**
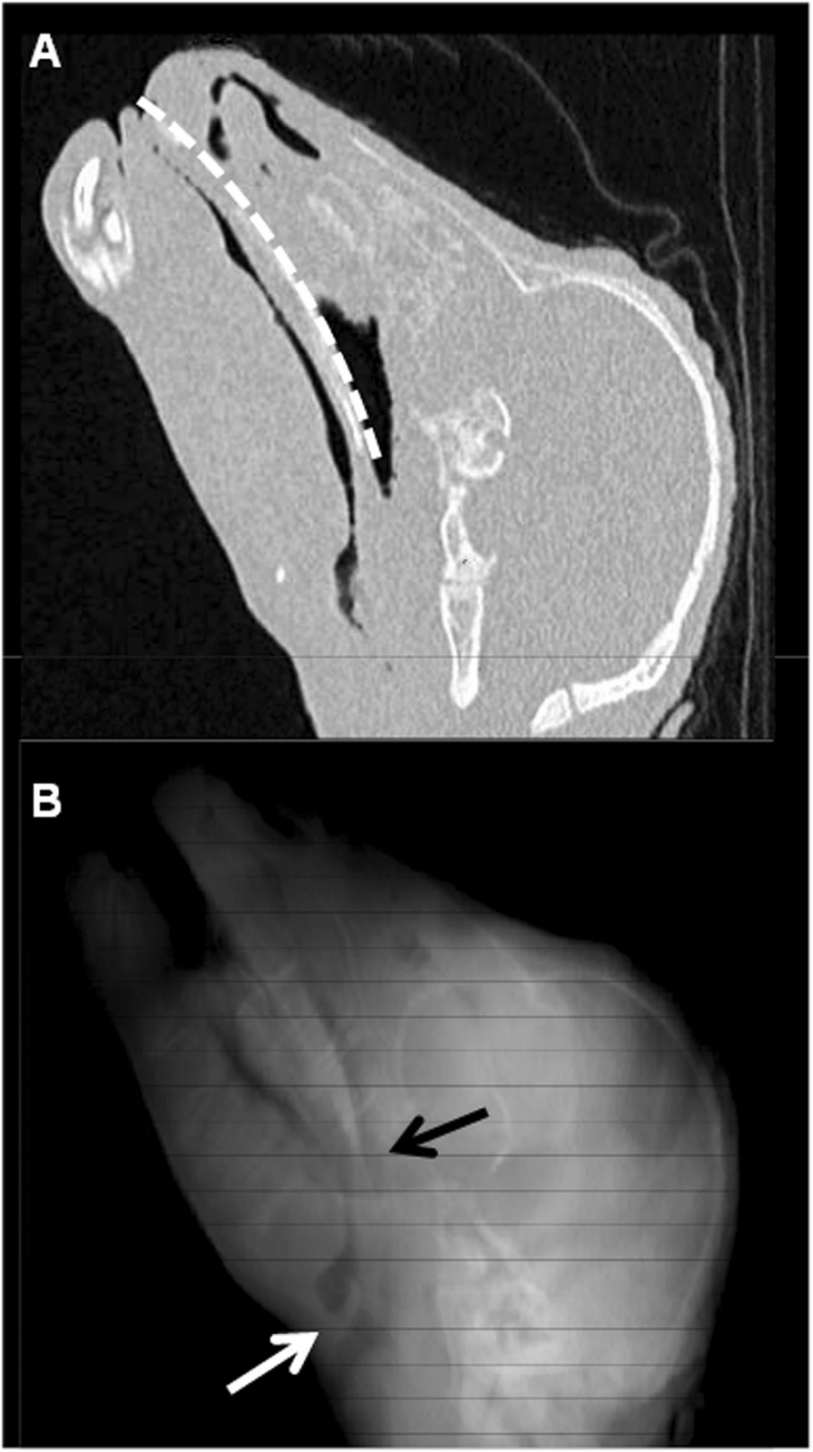
**Nasopharyngeal anatomy in the preterm lamb.** Computed tomographic images of the snout in a preterm lamb at 129 days gestation. **(A)** Parasagittal image showing dilatation of the nasal cavity deep within the nasopharynx. Path of nasal CPAP prong indicated by dotted white line (depth 6.5 cm). **(B)** 3D reconstruction of sagittal CT images; position of CPAP prong tip and epiglottis indicated by black and white arrows, respectively.

The CPAP delivery interface was a single nasal prong (shortened size 4.0 tracheal tube, Covidien, Mansfield, USA) positioned over a stylet to a depth of 6 to 7 cm [13], which based on anatomical studies resulted in a tip position around 2 to 3 cm proximal to the epiglottis (Figure 1). The choice of a single prong positioned deep in the nares was based on a) our previous experience of the failure to achieve an adequate seal with a close-fitting mask (G. Polglase, unpublished observations), b) the difficulties previously encountered in maintaining short binasal prongs in position [12] and c) preliminary studies in which we noted that a shallower single prong position did not transmit applied airway pressure adequately to the lung in the preterm lamb. Via the other nasal passage, a balloon-tipped catheter (size 10FG Foley catheter, Covidien) was placed in the oesophagus over a guide wire, positioned 6 cm below the cricoid cartilage, and the balloon was inflated with 3 mL saline to occlude the oesophagus. A 10FG Replogle suction tube (Covidien) was also passed transnasally with the tip immediately above the oesophageal balloon. The trachea was intubated with a cuffed size 4.0 endotracheal tube (ETT), and a surfactant delivery catheter was also placed in the trachea 9 cm below the vocal cords. After securing the tubes, the snout was wrapped with elastic bandage (Coban, 3 M, St. Paul, MN, USA) to seal the mouth.

With placental support maintained, the fetal chest was delivered, lung liquid was drained (expected recovery approximately 10 mL/kg) and respiratory inductance plethysmography (RIP) bands were placed around the chest and abdomen [14,15]. Recordings of RIP data, along with delivered airway pressure, were made continuously.

After cord clamping, the lamb was delivered, weighed and placed prone under a radiant warmer. A sustained inflation at 40 cm H_2_O pressure was immediately applied for 20 s via the ETT [16]. The ETT was immediately withdrawn from the trachea, without displacing the surfactant delivery catheter, clamped and left *in situ* in the oropharynx. Nasal IPPV was then commenced via the single prong, with initial positive end-expiratory pressure (PEEP) 8 cm H_2_O, peak pressure 30 cm H_2_O, inspiratory time 0.6 s, respiratory rate 30 bpm and FiO_2_ 0.4. We found, as have others [12,17], that a period of nasal IPPV was necessary to maintain gas exchange until spontaneous ventilation was established. Caffeine (20 mg/kg caffeine base) and doxapram (5 mg/kg) were administered to stimulate breathing [18], and a doxapram infusion was maintained at 2.5 mg/kg/h throughout.

Heart rate, blood pressure and oxygen saturation (SpO_2_) were monitored continuously, as was respiratory rate (RR) using RIP. Arterial blood gas analyses were performed periodically. Nasal IPPV rate was reduced as soon as practicable, with the goal of transitioning to support with CPAP alone within 30 min so long as respiratory rate and effort were maintained, with pH >7.20 and PaCO_2_ < 65. In the event of hypoventilation or apnoea, respiratory effort was promoted by cutaneous stimulation, repeated doxapram boluses (1 mg/kg, maximum three doses) and, if necessary, reinstitution of nasal IPPV (rate 2 to 30 bpm). FiO_2_ was adjusted throughout to maintain SpO_2_ 88% to 92% and PaO_2_ 50 to 80 mm Hg. CPAP/PEEP was increased (max 10 cm H_2_O) if FiO_2_ > 0.6 and decreased (minimum 6 cm H_2_O) if FiO_2_ < 0.25.

Body temperature was maintained at 38°C to 39°C. Infusions of 4% dextrose and 0.18% saline (intravenous) and heparinised saline (arterial) continued throughout. Measures to promote comfort included swaddling, intra-oral sucrose (2 mL prn) and tactile soothing, along with ketamine (0.1 mg/kg prn) if unsettled. Oropharyngeal, nasopharyngeal and oesophageal suction was performed at 15-min intervals.

Within 15 min of first transition to CPAP, all animals received surfactant (Curosurf 100 mg/kg) via the surfactant instillation catheter as part of a study investigating surfactant distribution, the results of which will be reported separately. Wherever possible, the study continued for 150 min after initiation of CPAP, after which a lethal dose of pentobarbital was administered.

### Data processing and analysis

Data recording, processing and analysis were undertaken using LabChart 7 (ADInstruments Pty Ltd, Bella Vista, Australia). For each animal, values for physiological parameters and arterial blood gas analyses were extracted 5 min after initiation of nasal IPPV (initial), immediately prior to commencement of CPAP (pre-CPAP), and at 15, 30, 45, 60, 90, 120 and 150 min after first trial of CPAP. At each time point, the ventilator set rate (if on NIPPV) was recorded, and the respiratory rate (mechanically assisted, spontaneous and total) was counted in a 2-min time window. Breaths were defined as any discrete positive deflection in chest and/or abdominal RIP voltage, and were counted using peak detection, with manual checking. Breaths were deemed to be mechanically assisted if occurring within 100 ms after onset of a positive pressure inflation. To overcome the limitations of sampling short periods of respiratory data, respiratory rate was also counted in the entire last hour of recording for each animal.

Data are presented as mean and standard deviation (SD) unless otherwise stated. Differences in parameters between time points were examined using repeated measures ANOVA. Dunnett’s *post hoc* test was used to compare values at all other time points with the pre-CPAP values.

## Results

Preliminary studies were performed in six animals (data not shown), in which the instrumentation techniques, approach to nasal IPPV and dosage of respiratory stimulants were refined. The study group consisted of ten lambs in which the experimental strategy thus developed was applied. An additional lamb in which prolonged need for resuscitation was followed by early pneumothorax and demise at 90 min was not included. Study animals were of gestation 132 ± 0.7 days (mean ± SD) and birth weight 3.6 ± 0.45 kg. Seven animals (70%) were from twin gestations, and three (30%) were male. The pre-delivery instrumentation procedure was performed without complication in all animals.

Transition from NIPPV to CPAP was first attempted at 28 ± 11 min of life. Duration of the experiment thereafter was 165 ± 20 min, with lambs supported on CPAP alone for 113 ± 48 min (68% ± 28% of time). In the final hour, lambs were on CPAP 79% ± 33% of the time. No lamb required endotracheal intubation; one lamb received NIPPV for all but a few minutes due to hypoventilation and was found to have a pneumothorax on *postmortem* examination. Data for this animal have been included in the analysis.

Most lambs showed the capacity for spontaneous respiration relatively soon after birth, which in some cases was masked by the use of NIPPV. Once on CPAP, apnoea events were common and were provoked in some cases by noxious stimuli including suctioning and instillation of surfactant. Beyond the initial loading dose and infusion, lambs received 1.6 ± 1.1 mg/kg doxapram as additional bolus doses (each 1 mg/kg) in order to prevent apnoea, with an apparent benefit in most cases. Transient myoclonus was observed in two lambs after repeated doxapram top-up dosing. As experience was gained, a clear benefit of reinstituting nasal IPPV at very low rate (2 to 5 bpm) in mitigating apnoea was noted (see below).

Physical activity was noted to gradually increase during the course of the experiment. Excess movement could generally be quelled with swaddling, tactile stimulation and oral sucrose. Most lambs spent long periods breathing comfortably on CPAP. Ketamine for apparent discomfort was administered to one animal.

**Figure 2:**
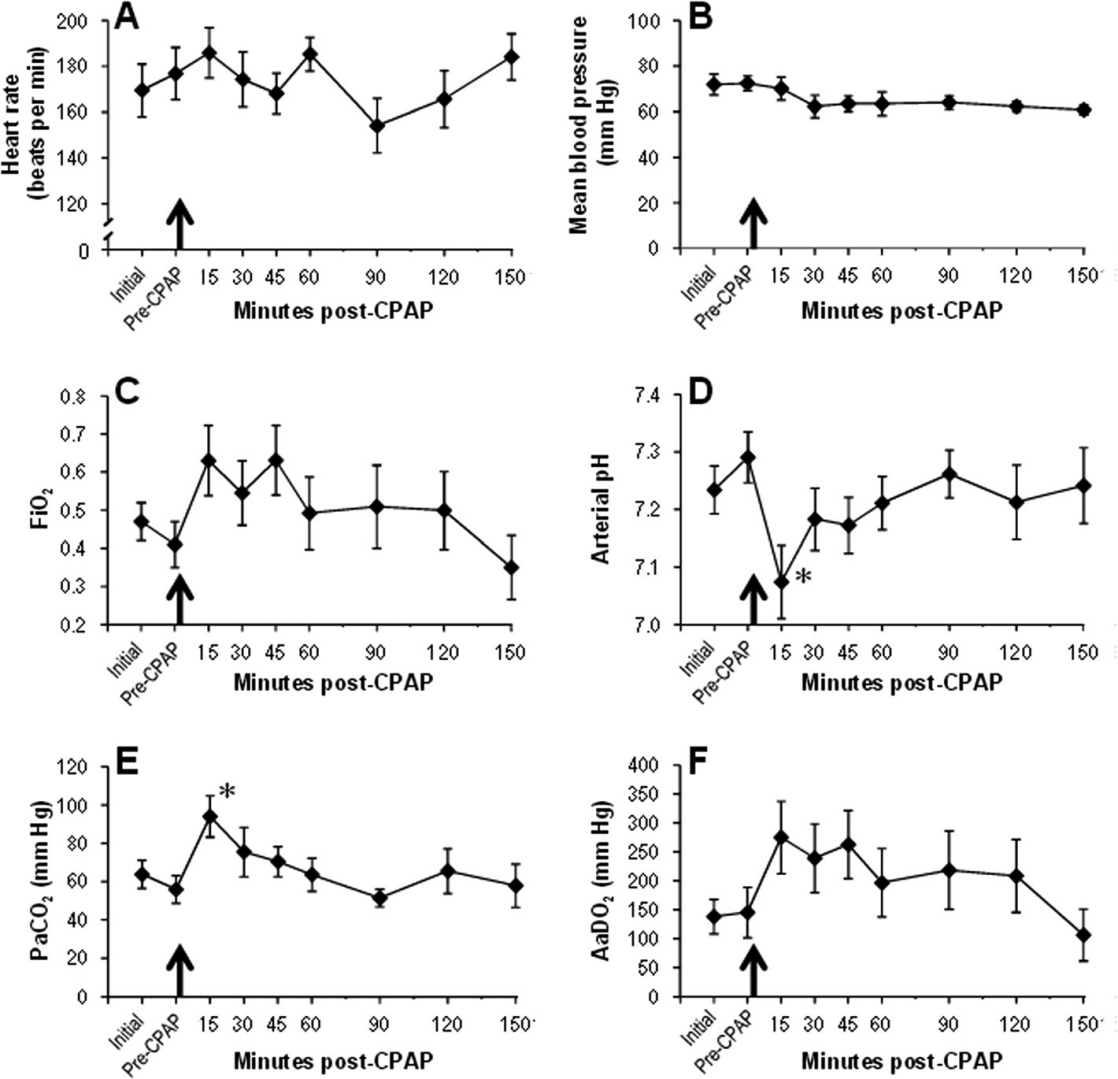
**Cardiorespiratory indices.** Mean and SE for cardiorespiratory indices at each experimental time point. Black arrow indicates the time of the first CPAP trial. **(A)** Heart rate, **(B)** mean blood pressure, **(C)** FiO_2_, **(D)** arterial pH, **(E)** PaCO_2_ and **(F)** AaDO_2_. *Differs from pre-CPAP value, *P* < 0.05, ANOVA with Dunnett’s *post hoc* test. CPAP, continuous positive airway pressure; AaDO_2_, alveolar-arterial oxygen difference.

Mean heart rate and blood pressure stabilised rapidly after delivery (Figure 2), were unaltered during transition to CPAP and remained stable thereafter. Gas exchange was readily achieved with nasal IPPV in early life. After attempted transition to CPAP, respiratory acidosis and increased oxygen requirement were transiently noted, although for all indices of gas exchange, the values at end-experiment were at or close to those achieved initially (Figure 2). The final blood gas analysis was performed on CPAP only (i.e. no NIPPV) in eight out of the ten animals.

**Figure 3:**
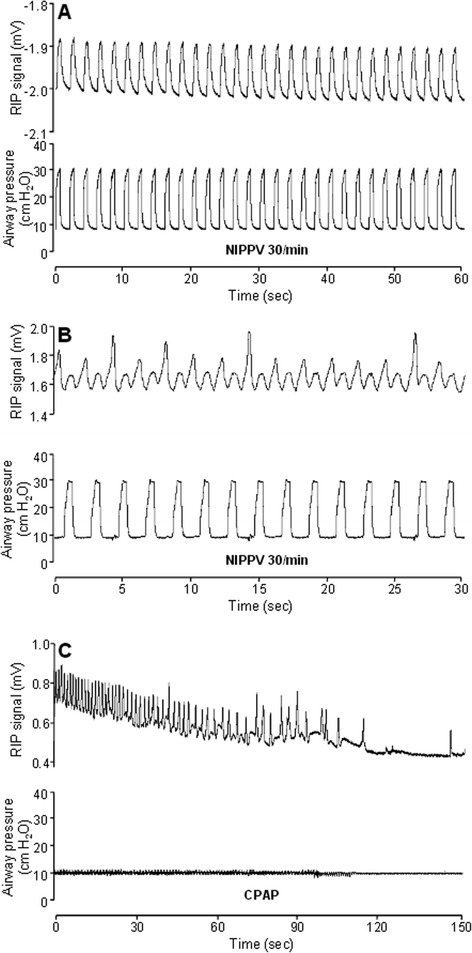
**Respiratory patterns (I).** Representative tracings of RIP voltage (top) and airway opening pressure (bottom) during the experiment. Note differing time scales between panels. **(A)** Lamb 5 at 5 min of life; full NIPPV support, no spontaneous breathing. **(B)** Lamb 5 pre-CPAP; on NIPPV, regular spontaneous breaths seen in addition to mechanically assisted breaths. **(C)** Lamb 8 soon after transition to CPAP; initially regular spontaneous breathing gradually slows to apnoea. RIP, respiratory inductance plethysmography; NIPPV, nasal intermittent positive pressure ventilation; CPAP, continuous positive airway pressure.

**Figure 4:**
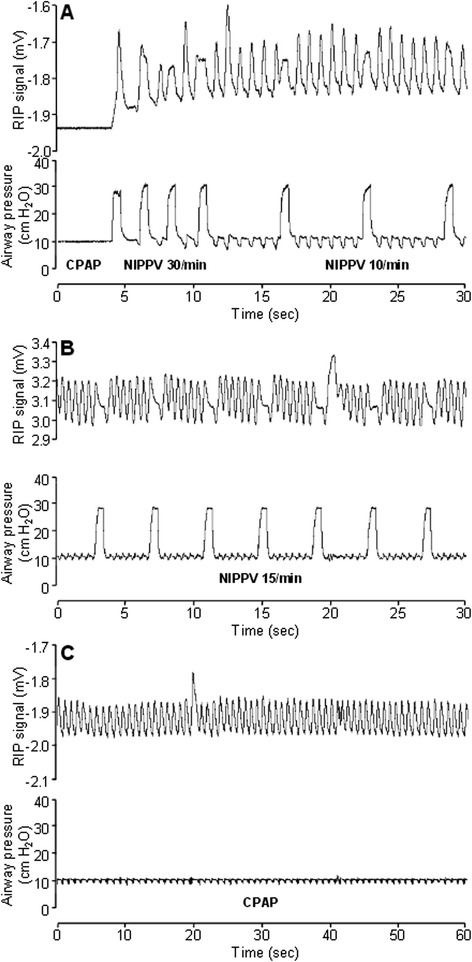
**Respiratory patterns (II).** Representative tracings of RIP voltage (top) and airway opening pressure (bottom) during the experiment. **(A)** Lamb 7 at 35 min after first attempt at CPAP transition; initial apnoea is followed by prompt return of spontaneous respiratory effort after reinstitution of NIPPV. **(B)** Lamb 8 at 30 min post-CPAP; NIPPV does not result in mechanically assisted breaths but does interfere with the cadence of spontaneous breathing. **(C)** Lamb 7 at 150 min; comfortably breathing on CPAP alone, spontaneous respiratory rate 65/min, PaCO_2_ 44 mm Hg and FiO_2_ 0.21. See Figure 3 legend for abbreviations.

Representative respiratory patterns during various phases of the experiment are shown in Figures 3 and 4, and summary data for NIPPV requirements and respiratory rates are shown in Table 1. Throughout the experiment, most NIPPV inflations resulted in an identifiable mechanically assisted breath (Figure 3A,B). When on CPAP, reinstitution of NIPPV with a low set rate was seen to stimulate spontaneous breathing in some cases (Figure 4A). Conversely, mechanical inflations appeared in some instances to interrupt the cadence of spontaneous breathing (Figure 4B). After the first attempt at transition to CPAP, spontaneous respiratory rate gradually increased and need for NIPPV decreased (Table 1). In the entire final hour of data recording, RR was 59 ± 14 breaths per minute, with 88% ± 26% of breaths being spontaneous and supported only with CPAP (Figure 4C).

**Table 1:** **NIPPV requirements and respiratory rates**

**Time**	**NIPPV used**	**Set NIPPV rate**	**Mechanically assisted RR**	**Spontaneous RR**	**Total RR**
	***n*** **(%)**	**(inflations/min)** ^**a**^	**(breaths/min)**	**(breaths/min)**	**(breaths/min)**
Initial	10 (100)	30 ± 0	25 ± 9.2	24 ± 24	50 ± 20
Pre-CPAP	10 (100)	30 ± 0	26 ± 8.9	21 ± 31	47 ± 25
15 min post-CPAP	5 (50)	15 ± 14	3.9 ± 5.9^b^	39 ± 30	43 ± 30
30 min post-CPAP	6 (60)	23 ± 11	14 ± 14	34 ± 30	48 ± 25
45 min post-CPAP	6 (60)	26 ± 10	14 ± 14	39 ± 32	52 ± 22
60 min post-CPAP	4 (40)	22 ± 20	8.8 ± 16^b^	41 ± 30	50 ± 22
90 min post-CPAP	3 (30)	33 ± 6.0	8.9 ± 14	49 ± 30	58 ± 25
120 min post-CPAP	1 (10)	46 ± 0	4.6 ± 15^b^	55 ± 21^b^	60 ± 20
150 min post-CPAP	2 (20)	31 ± 1.4	4.7 ± 10^b^	56 ± 24^b^	61 ± 15

Values for NIPPV requirements and respiratory rates at each time point; mean ± SD unless stated. Respiratory rate (RR) counted in a 2-min window; reported values are for all lambs, whether or not receiving NIPPV. See text for definition of mechanically assisted breath. ^a^Reported value for set NIPPV rate includes only lambs receiving NIPPV. ^b^Differs from pre-CPAP value, *P* < 0.05, ANOVA with Dunnett’s *post hoc* test.

## Discussion

In this study, we sought to develop a robust model of nasal CPAP in preterm lambs 131 to 133 days gestation. We found that following initial use of NIPPV, lambs could be stabilised on nasal CPAP in the first hour of life, with a transient respiratory acidosis that resolved as spontaneous respiratory activity increased. Most lambs were breathing comfortably on CPAP during the last hour of the experiment.

The use of nasal IPPV appears to be a very effective means of transitioning the non-intubated preterm lamb to CPAP in the first hour of life. The deep nasal tube and relatively leak-free experimental setup meant that the nasal positive pressure inflations regularly resulted in appreciable chest excursion, in contrast to what is often seen in human infants [19]. This in turn allowed sufficient ventilation to avoid early respiratory acidosis, with PaCO_2_ values during NIPPV slightly better than those previously noted with nasal high-frequency ventilation in preterm lambs of similar gestation [13]. In some lambs, NIPPV appeared to be a potent stimulus of spontaneous respiratory effort, possibly with a mechanism similar to Head’s paradoxical reflex [20]. This was exploited by the use of low NIPPV rates (2 to 5 bpm) in several instances where there was recurrent apnoea on CPAP alone.

Transition to CPAP at around 30 min of life was associated with a significant but short-lived respiratory acidosis and increase in oxygen requirement. This brief deterioration in gas exchange did not appear to be ultimately problematic, and there were no substantial heart rate or blood pressure changes as CPAP was established. Most animals were on CPAP throughout the last hour of the experiment. Gas exchange at end-experiment was satisfactory, with PaCO_2_ and AaDO_2_ values on par with pre-CPAP readings and also with values previously noted in intubated lambs on CPAP [8]. A longer period of CPAP, and eventually weaning from CPAP, would have been possible in nine out of the ten animals, the exception being the lamb with untreated pneumothorax, which had worsening acidosis and poor oxygenation at end-experiment. Note that pneumothorax is not an unexpected outcome in a preterm CPAP model, being seen in as many as 9% of preterm infants on CPAP [21].

The use of caffeine and doxapram may have aided in the stimulation of regular respiratory effort in our CPAP model. Both are effective for this purpose in the premature lamb [22] and may have a synergistic effect when used together [18]. Doxapram is used in veterinary medicine as a treatment for apnoea in preterm and asphyxiated animals, with a relatively wide gap between therapeutic and toxic windows [23]. Nevertheless, some unwanted neurological effects, in particular myoclonic activity, were seen after bolus doses in some animals, emphasising the need for judicious use of this medication in this setting.

The oesophageal balloon was an effective means of preventing gaseous abdominal distension, previously a limiting factor to the development of a CPAP model in the non-intubated lamb [12], and a regular complication of CPAP in preterm infants [24]. The occlusion of the oesophagus was probably of most value during NIPPV, during which significant gastric distension may occur, especially with a long nasal tube [25].

The effective transition to CPAP via NIPPV and the relative ease of ventilation thereafter suggest that this CPAP model may be applicable in even less mature preterm lambs. Below 130 days gestation, the disturbances of the surfactant system of the lamb more faithfully replicate the surfactant deficiency seen in very preterm infants. At least in intubated lambs, the maturity of the surfactant system has been found to be an important factor in determining whether transition to CPAP is possible [6]. This observation can potentially now be re-examined in a non-intubated preterm lamb using less invasive methods of surfactant delivery [2,3].

Several limitations of our study of the preterm lamb on CPAP are evident. Animals were in a narrow range of gestation, and all were managed with the same treatment protocol, including administration of surfactant. Respiratory support only continued for 150 min after CPAP, and no attempt at weaning from CPAP was made. Finally, we have not made any physiological or histological comparison with lambs intubated and ventilated from the outset. These studies, and many others examining different research questions related to the premature infant on NIPPV and CPAP, should be possible with this model.

## Conclusions

In conclusion, with the use of nasal IPPV, oesophageal occlusion and respiratory stimulants, non-intubated very preterm lambs can be effectively transitioned to nasal CPAP soon after birth. This model will be valuable for further research investigating non-invasive respiratory support after premature birth.

## Abbreviations

CPAP: continuous positive airway pressure
ETT: endotracheal tube
NIPPV: non-invasive positive pressure ventilation
PEEP: positive end-expiratory pressure
RIP: respiratory inductance plethysmography
RR: respiratory rate
SpO_2_: oxygen saturation
